# Taxonomic composition and carbohydrate-active enzyme content in microbial enrichments from pulp mill anaerobic granules after cultivation on lignocellulosic substrates

**DOI:** 10.3389/frmbi.2023.1094865

**Published:** 2023-09-27

**Authors:** Mabel T. Wong, Camilla L. Nesbø, Weijun Wang, Marie Couturier, Vincent Lombard, Pascal Lapebie, Nicolas Terrapon, Bernard Henrissat, Elizabeth A. Edwards, Emma R. Master

**Affiliations:** ^1^Department of Chemical Engineering and Applied Chemistry, University of Toronto, Toronto, ON, Canada; ^2^Université Grenoble Alpes, Centre National de la Recherche Scientifique (CNRS), Centre de Recherches sur les Macromolécules Végétales (CERMAV), Grenoble, France; ^3^Aix-Marseille Université, Centre National de la Recherche Scientifique (CNRS), UMR7257, Laboratoire Architecture et Fonction des Macromolécules Biologiques (AFMB), Marseille, France; ^4^L’Institut National de Recherche our l’Agriculture, l’Alimentation et l’Environnement (INRAE), USC1408 Laboratoire Architecture et Fonction des Macromolécules Biologiques (AFMB), Marseille, France; ^5^Department of Biological Sciences, King Abdulaziz University, Jeddah, Saudi Arabia; ^6^Technical University of Denmark, Danmarks Tekniske Universitet (DTU) Bioengineering, Lyngby, Denmark; ^7^Department of Bioproducts and Biosystems, Aalto University, Espoo, Finland

**Keywords:** carbohydrate active enzymes, metagenomics, lignocellulose, anaerobe, bioconversion

## Abstract

Metagenomes of lignocellulose-degrading microbial communities are reservoirs of carbohydrate-active enzymes relevant to biomass processing. Whereas several metagenomes of natural digestive systems have been sequenced, the current study analyses metagenomes originating from an industrial anaerobic digester that processes effluent from a cellulose pulp mill. Both 16S ribosomal DNA and metagenome sequences were obtained following anaerobic cultivation of the digester inoculum on cellulose and pretreated (steam exploded) poplar wood chips. The community composition and profile of predicted carbohydrate-active enzymes were then analyzed in detail. Recognized lignocellulose degraders were abundant in the resulting cultures, including populations belonging to Clostridiales and Bacteroidales orders. Poorly defined taxonomic lineages previously identified in other lignocellulose-degrading communities were also detected, including the uncultivated Firmicutes lineage *OPB54* which represented nearly 10% of the cellulose-fed enrichment even though it was not detected in the bioreactor inoculum. In total, 3580 genes encoding carbohydrate-active enzymes were identified through metagenome sequencing. Similar to earlier enrichments of animal digestive systems, the profile encoded by the bioreactor inoculum following enrichment on pretreated wood was distinguished from the cellulose counterpart by a higher occurrence of enzymes predicted to act on pectin. The majority (> 93%) of carbohydrate-active enzymes predicted to act on plant polysaccharides were identified in the metagenome assembled genomes, permitting taxonomic assignment. The taxonomic assignment revealed that only a small selection of organisms directly participates in plant polysaccharide deconstruction and supports the rest of the community.

## Introduction

Wood and agricultural fibre comprise cellulose, hemicelluloses and lignins (i.e., lignocellulose) and represent major resources for the production of renewable fuels, chemicals and materials. Biological deconstruction of lignocellulose is catalyzed by the concerted action of carbohydrate-active enzymes (CAZymes) and metagenomes of lignocellulose-degrading communities are an especially rich source of CAZymes that could be used for lignocellulose processing (Terrapon et al., 2018; Garron and Henrissat, 2019; Drula et al., 2022).

Metagenomic studies aimed at identifying new CAZymes have sequenced grass-feeding gut microbiota (Al-Masaudi et al., 2017; Deusch et al., 2017; Wang et al., 2019) and wood degrading gut microbiomes of termites (Warnecke et al., 2007; Liu et al., 2019; Romero Victorica et al., 2020), beetle (Scully et al., 2013), wood wasp (Adams et al., 2011) moose (Svartström et al., 2017; Wong et al., 2017), and beaver (Wong et al., 2017; Armstrong et al., 2018). Most pertinent CAZymes for lignocellulose processing can be uncovered through sequencing metagenomes of enrichment cultures (Jimenéz et al., 2016; Wang et al., 2016; Schultz-Johansen et al., 2018; Tomazetto et al., 2020; Borjigin et al., 2022). For instance, in our previous study of digestive microbiomes from Canadian beaver (*Castor canadensis*) and North American moose (*Alces americanus*), we report substrate-induced convergence of taxonomic profiles and CAZyme compositions following anaerobic cultivation of corresponding inocula on cellulose or pretreated wood fibre.

Large-scale bioreactor systems that process industrial and municipal lignocellulosic materials represent additional, compelling sources of CAZymes (Wilkens et al., 2017). Besides municipal bioreactors, anaerobic digesters that transform pulp mill effluent to biogas are an untapped source of enzymes for lignocellulose processing. In most cases, pulp mill effluent is mechanically processed to remove suspended solids and then biologically treated through an aerobic activated sludge process (Thompson et al., 2001). The generated biosludge is subsequently dewatered and incinerated for power generation or else landfilled. Increasingly, anaerobic bioconversion of pulp mill effluent prior to aerobic treatment is employed to reduce the accumulation of secondary biosludge while also generating biogas (Hagelqvist, 2013; Meyer and Edwards, 2014). About 10% of pulp mills worldwide have installed anaerobic treatment technologies, and in particular, internal circulation reactors (Meyer and Edwards, 2014). These high-rate reactors enable effective bioconversion of organic compounds into biogas via the sequential activity of hydrolytic bacteria, acetogens, and methanogenic archaea (Tauseef et al., 2013; Kamali et al., 2016).

Herein, we investigated whether microbial communities enriched from pulp mill anaerobic bioreactors encode an assemblage of CAZymes distinct from those identified through metagenomic analysis of natural digestive systems. To address this question, microbial granules were collected from an anaerobic internal circulation bioreactor located at a pulp mill and enriched for three years on multiple lignocellulosic carbon sources. Subsequent metagenome sequencing and metagenome assembled genomes (MAGs) permitted CAZyme assignment to specific microbial species originating from the pulp mill bioreactor. The current study underpins the significance of both environmental inoculum and enrichment condition on the taxonomic distribution and functional potential of resulting microbial communities.

## Materials and methods

### Collection of pulp mill anaerobic granules

Anaerobic granules were collected in 2009 from an internal circulation bioreactor located at a pulp mill in Québec, Canada. The bioreactor typically received 15,000 m^3^/day of mixed wastewater including acid condensate from the evaporator system and bleached chemi-thermomechanical pulp effluent.

### Set up and maintenance of lignocellulose-degrading enrichment cultures from pulp mill anaerobic granules

The anaerobic granules were used as inoculum for enrichment cultures grown at 36 °C under anaerobic conditions as previously described (Wong et al., 2016). Briefly, sulphide-reduced mineral medium (pH 7.0) was prepared and purged with 80% N_2_, 20% CO_2_ gas mixture (Wong et al., 2017). Approximately 15 mL of anaerobic granules were transferred to 160 mL Wheaton glass serum bottles amended with a lignocellulosic substrate (average 36.1 mg chemical oxygen demand (COD) equivalent per bottle) and 45 mL of mineral medium. The lignocellulosic amendments were i) microcrystalline cellulose (Avicel PH101, Sigma-Aldrich, MO, USA), ii) cellulose + lignosulphonate, iii) cellulose + tannic acid (Sigma-Aldrich, MO, USA), and iv) steam-exploded poplar (SunOpta Inc., Canada). Biogas production was regularly monitored using a pressure transducer (Omega PX725 Industrial Pressure Transmitter, Omega DP24-E Process Meter). When biogas production ceased, the microbial community was transferred to a new bottle with fresh anaerobic medium and lignocellulose carbon source (Wong et al., 2016). This process was repeated eight times over a period of 3 years prior to DNA extraction and sequencing.

### DNA extraction and sequencing

DNA was extracted from each culture after three years of enrichment on respective substates. Samples (10 mL) were taken just as biogas production began to slow down and centrifuged at 15,000 x g for 15 min at 4 °C. Total community DNA was then extracted from the resulting pellets using the QIAamp DNA Stool Mini Kit. The concentration and quality of the extracted DNA was assessed by measuring the 260/280 absorbance ratio using a Nanodrop 2000 spectrophotometer (Thermo Scientific, MA, United States) before storing the DNA samples at -80 °C. The DNA samples were used for 16S rRNA gene amplicon sequencing and for metagenomic shotgun sequencing (Wong et al., 2016). The V6-8 hypervariable region of 16S rRNA genes was amplified with 926 Forward (5’- AAACTYAAAKGAATTGACGG) and 1392 Reverse (5’- ACGGGCGGTGTGTRC) primers and multiplexed with 10-nt Roche barcodes by polymerase chain reaction (**Table S1**; DeAngelis et al., 2012). Multiplex-pyrosequencing was performed in 2013 on a 454 GS FLX platform (454 Life Sciences-a Roche Company, Branford, CT, USA) at the Génome Québec Innovation Centre. Illumina paired-end sequencing with TruSeq library was performed in 2015 using a Illumina HiSeq 2000 (Illumina Inc., San Diego, CA, USA) at the Génome Québec Innovation Centre.

### Analyses of 16S rRNA gene pyrosequences

Pyrosequencing output was converted to sequence reads and quality scores using Roche 454 Life Science propriety software (http://www.454.com) and then analyzed by QIIME 1.8.0 (Caporaso et al., 2010) as previously described (Wong et al., 2016). Combining the taxonomic profiles from our previous analysis on lignocellulose-degrading microcosms from beaver dropping and moose rumen (Wong et al., 2017), relative abundances of genera representing ≥ 1% for at least one of the samples were extracted for hierarchical clustering (correlation clustering and average linkage) and Principal Component Analysis using R statistics in ClustVis (Metsalu and Vilo, 2015). Non-parametric Kruskal-Wallis test was conducted using R script (R Development Core Team, 2010).

### Metagenome assembly, annotation of CAZyme families, multi-modular sequences, and polysaccharide utilization loci

Quality trimming and assembly of metagenomics shotgun sequences were first done using Abyss v 1.3 (Simpson et al., 2009) as described in Wong et al., 2017. In addition, the raw reads (70,817,105 pairs for cellulose-fed enrichments, and 78,824,461 pairs for pretreated poplar-fed enrichments) were quality trimmed and assembled using the Anvi’o v6.2 snakemake metagenomic workflow (Eren et al., 2021). This pipeline uses the read quality control methods developed in Minoche et al., 2011. Three assemblers were used; megahit v.1.1.2 (Li et al., 2015), spades v. 3.12.0 (metaspades mode, Nurk et al., 2017), idba v. 1.1.3 (Peng et al., 2012), and the assemblies were performed both on the samples separately and combined in co-assemblies. The assemblies were binned into metagenome assembled genomes (MAGs) using metabat2 (Kang et al., 2019) and maxbin2 (Wu et al., 2016), and the resulting MAGs were compared and dereplicated using dRep (Olm et al., 2017) with a pairwise ANI cut-off at 99%, % completion > 75% and contamination < 25%. The resulting collection of MAGs were combined into one dataset; reads were mapped back to the MAGs using bowtie2 v 2.4.2 to obtain coverage information and visualized in Anvi’o. Taxonomic classification was assigned to each MAG using the GTDB-TK tool kit which compares the genomes to the genome taxonomy database (Chaumeil et al., 2020). Taxonomic composition of metagenomic reads based on rRNA reads extracted from the libraries and classified to order level was performed using the PhyloFlash v. 3.4 (Gruber-Vodicka et al., 2020) software and the SILVA v.138 database.

Prediction of open reading frames, CAZyme families, PULs as well as taxonomic assignment of the predicted CAZymes were performed on the Abyss-assembly and visualized in accordance to the methodology described in Wong et al., 2017. Count of identified sequences in a given CAZyme family was normalized by the number of open reading frames. Normalized count of predicted plant polysaccharide-active CAZyme families from the earlier beaver dropping and moose rumen metagenomes (Wong et al., 2017) were combined with the current dataset for hierarchical clustering (correlation clustering and average linkage) and PCA using R statistics in ClustVis (Metsalu and Vilo, 2015). The identified genes encoding CAZymes were mapped to the MAGs using BLASTN.

## Results and discussion

### Establishment of biogas-producing microbial enrichments

**Figure 1:**
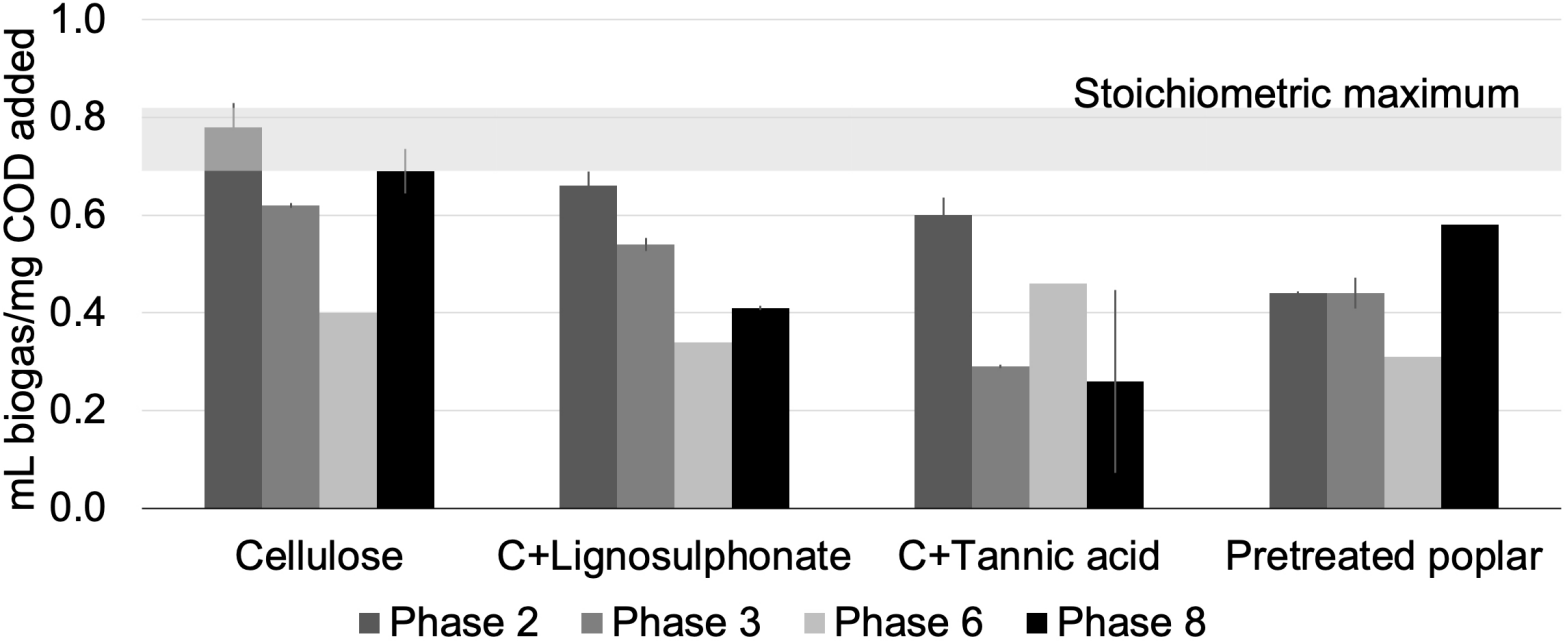
Biogas production by microcosms fed with lignocellulosic carbon sources over 3-years. DNA was extracted from Phase 8 for this study. The estimated stoichiometric maximum biogas yield is shown by the horizontal grey band to provide a reference for the extent of conversion of each substrate (Symons and Buswell, 1933; Wong et al., 2016); error bars indicate standard deviation; *n* = 3.

Anaerobic enrichment cultures were established and biogas production was sustained over three years on four lignocellulosic carbon sources. As observed previously (Wong et al., 2016), biogas yield per COD added dropped during early stages of enrichment, likely due to depletion of readily digestible COD present in the inoculum (**Figure 1**). The subsequent increase in biogas yield was consistent with microbial acclimatization to the amended carbon sources. Following the three year acclimatization period (i.e., by the eighth transfer), biogas yields were highest for cellulose-fed cultures (0.69 mL biogas/mg COD added), followed by pretreated poplar (0.58 mL biogas/mg COD added), cellulose + lignosulphonate (0.41 mL biogas/mg COD added), and cellulose + tannic acid (0.15 mL biogas/mg COD added). The apparent inhibition of biogas production in cultures amended with tannic acid was also observed in enrichment cultures from beaver droppings and moose rumen (Wong et al., 2016).

### Microbial diversity in lignocellulose-degrading microcosms enriched from pulp mill anaerobic granules

**Figure 2:**
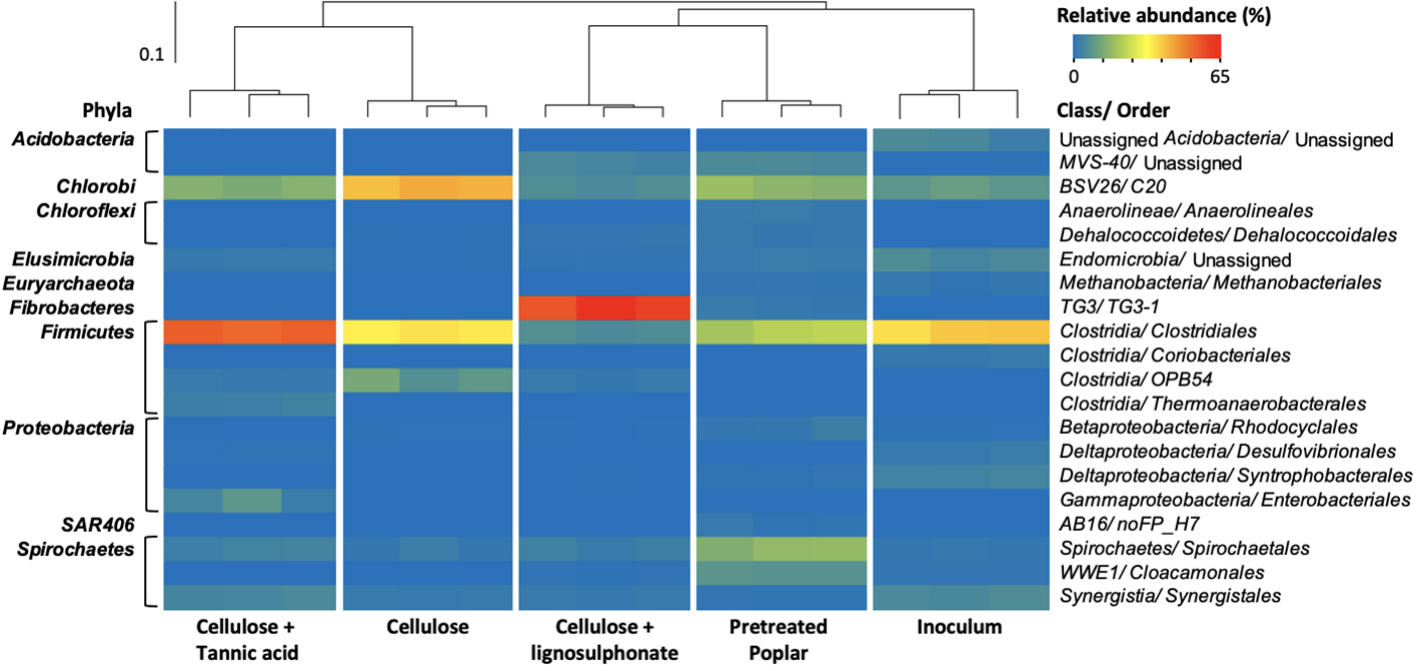
Heatmap of the microbial orders (≥ 1% in at least one sample) identified by 16S rRNA analysis of pulp mill anaerobic granules and corresponding enrichment cultures, with the sample similarities calculated by weighted UniFrac method and summarized by UPGMA clustering.

A total of 99,218 high-quality 16S rRNA gene pyrosequences from the established anaerobic enrichments were assigned to 5,359 OTUs at 97% similarity threshold (**Table S2A**). Beta-diversity based UPGMA (unweighted pair group method with arithmetic mean) clustering of the microbial communities revealed two main clades: i) microcosms fed with cellulose or cellulose + tannic acid, and ii) inoculum and microcosms fed with cellulose + lignosulphonate and pretreated poplar (**Figure 2**). The clustering of biological replicates for a given enrichment further reflected the statistical differences between the communities following the amendments, as well as the reproducibility of enrichments and 16S rRNA gene analysis. The following paragraph highlights the relative abundances of main microorganisms in each culture to uncover shifts in populations that correlate with the amended substrate.

Prior to enrichment, pulp mill anaerobic granules were dominated by *Clostridiales* (~ 40%), *Bacteroidales* (10%), *Synergistales* (6%), *Anaerolineales* (6%), *Syntrophobacterales* (5%) bacterial orders, as well as methanogens belonging to *Methanosarcinales* order (5%) (**Figure 2**; **Table S2B**). The species richness of the microbial communities decreased upon anaerobic enrichment on cellulose, and to a lesser extent, pretreated poplar (**Figure S1**). Nevertheless, lineages recognized as lignocellulose degraders were consistently abundant in all enrichment cultures. For example, populations belonging to *Clostridiales* and *Bacteroidales* orders represented more than 75% of the microbial community in the cellulose-fed enrichments and remained ~ 40% in those fed with pretreated poplar (**Table S2B**). Moreover, the uncultivated Firmicutes lineage *OPB54* represented up to 10% of the cellulose-fed enrichment and ~ 3% in all other enrichments even though it was not detected in the anaerobic granule inoculum (**Table S2B**). Members of *OPB54* are reported in diverse ecologies including the gut microbiomes of beaver and moose (Wong et al., 2017) and can ferment a wide variety of carbohydrates (Liu et al., 2014). Similarly, the ‘termite group 3’ (*TG3*) class of the *Fibrobacterota* phylum was abundant (60%) in cultures enriched on cellulose + lignosulphonate even though it was not detected in the inoculum. As its name implies, the *TG3* class was initially detected in termite guts (Hongoh et al., 2006) and later identified in diverse habitats, including anaerobic digesters (Rahman et al., 2016).

### Metagenome sequencing and assembly

The cellulose-fed and pretreated poplar-fed enrichments exhibited highest biogas yields compared to the other enrichments generated herein and so corresponding metagenomic DNA was collected for sequencing and assembly. The metagenomic DNA from cellulose-fed and pretreated poplar-fed enrichments yielded 64 and 68 million high quality reads, respectively. The taxonomic composition of the metagenomes was assessed by extracting rRNA genes and comparing them to the SILVA database. The taxonomic composition at order level was similar to the community structure obtained from the amplicon data (**Figure S2**), with *Clostridiales* and *Bacteroidales* representing > 75% of the microbial community in the cellulose-fed enrichments and ~30% in those fed with pretreated poplar.

The metagenome reads were assembled using four assemblers; assembly statistics are provided in **Table S3**. The assembled contigs were binned into 2,417 bins, which were dereplicated using a 99% identity cut-off to 139 unique metagenome assembled genomes (MAGs) with completeness > 75% and contamination < 25% as determined by CheckM (Parks et al., 2015; **Table S4**). Of these, 127 MAGs had at least medium quality with less than 10% contamination (Bowers et al., 2017). Moreover, 83% of reads from cellulose-fed enrichments and 75% of reads from pretreated poplar-fed enrichments were mapped to the MAGs.

**Figure 3:**
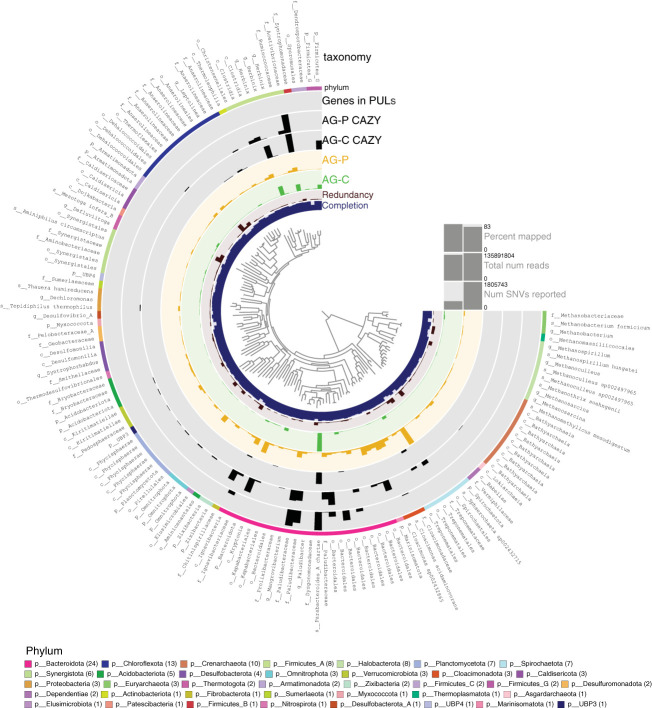
Metagenome Assembled Genomes (MAGs) from anaerobic granules (AG) following enrichment on cellulose (AG-C) or pretreated poplar (AG-P). The 139 dereplicated MAGs from the AG-C and AG-P metagenomes are visualized using the Anvi’o software (Eren et al., 2021). The phylogenetic tree at the centre of the figure is a maximum likelihood tree based on concatenated ribosomal proteins calculated using FastTree (Price et al., 2010). From inner to outer ring: The first ring shows the % completion of each MAG calculated based on single-copy core genes (SCGs) with maximum set to 100%. The second ring indicates the level of redundancy (or possible contamination) also based on SCGs with maximum set to 30%. The green and yellow bar for each MAG shows the mean coverage of each MAG in each sample, with maximum at 1284 and 587 for AG-C and AG-P, respectively. The AG-C CAZY and AG-P CAZY bar shows number of blast matches to carbohydrate-active enzyme sequences predicted to act on plant-polysaccharides, where the maximum number in each case is 79 and 80, respectively (pairwise sequence identity was 99%-100%). Genes in PULSs corresponds to the number of CAZymes encoded in MAGs that are predicted to act on plant polysaccharides and be localized in polysaccharide utilization loci (PULs). The outer circle shows the taxonomic domain by color, predicted by Anvio using the genome taxonomy database (gtdb, https://gtdb.ecogenomic.org/). Finally, the best taxonomic classification of each MAG is provided. A ‘p’ in front of the taxon name indicates this is a phylum classification, a ‘c’ indicates classification to class, ‘f’ indicates family level classification, ‘o’ indicates order level classification, ‘g’ indicates classification to genus and ‘s’ indicates species level classification. The bars on the right-side shows percent and total number of reads mapped to the MAGs from each metagenome. SNV denotes single nucleotide variants and shows the level of diversity in all the MAGs when reads from the two metagenomes are mapped back to the best MAG.

Taxonomy and abundances of each MAG are shown in **Figure 3** and **Table S4**. Most abundant MAGs obtained from the cellulose-fed enrichment were classified as belonging to *Parabacteroides* (TG_idbaud_maxbin.144), *Herbinix* (TGCmsp_mb_bin.66) and *Acetivibrionaceae* (TGPidbaud_mb_bin_32). By comparison, the community structure was more even in the pretreated poplar-fed enrichment, and most abundant MAGs were classified to *Treponemataceae* Spiro-10 (TGP_msp_maxbin.003), *Bacteroidota* UBA10030 (TGP_msp_maxbin.004) and *Cloacimonas* sp. 002432865 (TGP_mgh_maxbin.005). One abundant MAG shared by the two metagenomes at the 99% identity was the *Acetivibrionaceae* MAG TGPidbaud_mb_bin_32. However, inspection of reads mapped to this MAG revealed that each enrichment contains a distinct, but highly similar strain of this taxon.

### CAZyme family profiles of the cellulose- and poplar-fed microcosms

In total, 3,580 genes encoding 95 families of glycoside hydrolases (GHs), 12 families of carbohydrate esterases (CEs), 13 families of polysaccharide lyases (PLs), 32 families of glycosyltransferases (GTs) and 33 families of carbohydrate binding modules (CBMs) were predicted from the two metagenomes (**Table S5**). This corresponded to approximately 3% of open reading frames predicted in the cellulose-fed and pretreated poplar-fed enrichments. No auxiliary redox enzymes were identified as expected for anaerobic microbial communities.

**Figure 4:**
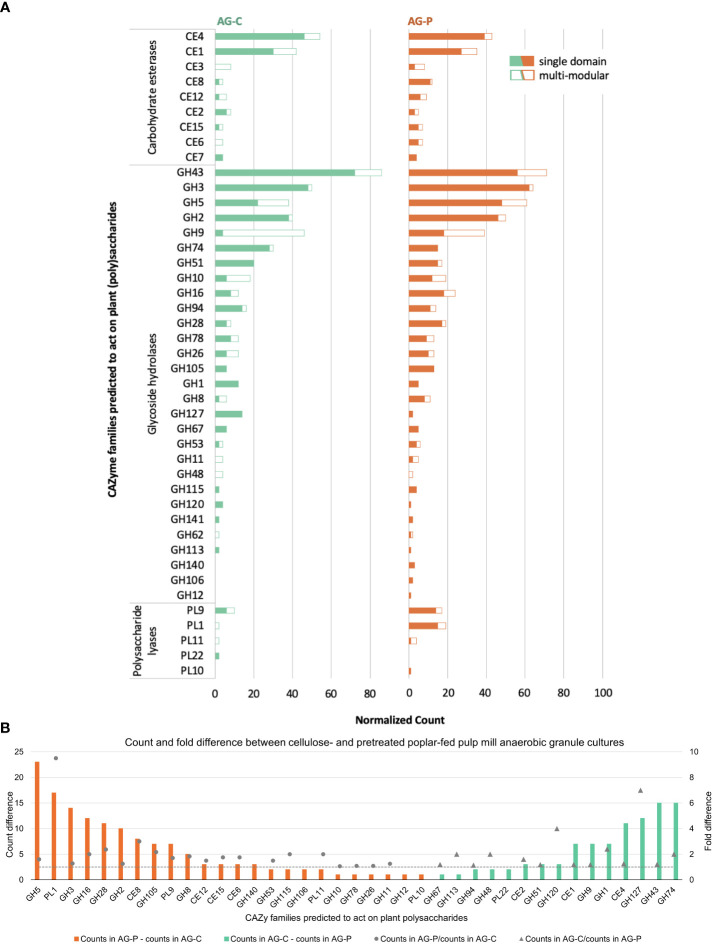
**(A)** Distribution of plant polysaccharide degrading CAZyme families as single and multi-modular domains. **(B)** Normalized count and fold difference of CAZyme families predicted to act on plant polysaccharides between pretreated poplar- and cellulose-fed pulp mill anaerobic granule cultures. Fold difference was only calculated for non-zero counts.

Among the predicted CAZymes, 245 genes from the cellulose-fed enrichment and 555 from the pretreated poplar-fed enrichment were predicted to be involved in plant polysaccharide deconstruction (**Figure 4**). Similar to cellulose-fed and pretreated poplar-fed enrichments of moose rumen samples and beaver droppings (Wong et al, 2017), nearly 30% of these CAZymes belonged to families GH2, GH3, GH5, GH9, GH43, CE1, and CE4 (**Figure 4A**). Thus, a similar CAZyme profile emerged from industrial and natural digestive systems following their enrichment on the same cellulosic and pretreated-poplar substrates. Closer inspection of the CAZyme profiles reported herein, however, distinguished the metagenome of the pretreated poplar-fed enrichment from the cellulose counterpart by its higher occurrence of CAZyme families predicted to act on pectin, including more than nine times the number of sequences belonging to family PL1 (pectate lyases), and more than twice the number of sequences belonging to families CE8 (pectin methylesterase), GH28 (polygalacturonases) and GH105 (unsaturated glucuronyl/galacturonyl hydrolases) (**Figure 4B**).

The majority (> 93%) of the predicted CAZymes acting on plant polysaccharides were included in the MAGs constructed from the metagenomes (**Figure 3**, **Table S6**). MAGs from cellulose-fed enrichments that carry the highest number of predicted CAZymes targeting plant polysaccharides were also the most abundant in the metagenome; however, this was not the case for pretreated poplar-fed enrichments (**Tables S6**, **S7**). For example, the most abundant MAG in the cellulose-fed enrichment is classified as *Acetivibrionaceae* (TGPidbaud_mb_bin_32) and carries 79 of the 245 genes from the corresponding metagenome that are predicted to encode CAZymes acting on plant polysaccharides. The MAG belonging to *Acetivibrionaceae* from the pretreated poplar-fed enrichment also encoded the highest number of CAZymes predicted to deconstruct plant polysaccharides; however, this MAG was low in abundance (**Tables S6**, **S8**). Instead, the most abundant MAGs in the pretreated poplar-fed enrichment were the *Bacteroidota* UBA10030 MAG (TGP_msp_maxbin.004) that carried only 61 CAZymes predicted to act on plant polysaccharides, *Treponemataceae* Spiro-10 MAG (TGP_msp_maxbin.003) that carried only 17 and the *Cloacimonas* sp. 002432865 MAG (TGP_mgh_maxbin.005) that carried none. Notably, the *Bacteroidota* MAG is distinguished by having a comparatively high content of predicted polysaccharide lyases and pectinases belonging to family GH28.

As mentioned in the above section, both *OPB54* and *TG3* species were identified in the enrichment cultures but not the anaerobic granule inoculum, and corresponding OTUs were previously reported in other lignocellulose-degrading communities. Herein, a MAG corresponding to the *OPB54* species was identified in the cellulose-fed enrichment (TGC_mgh_maxbin.005), which is assigned to the family UBA8346 that forms a novel Firmicutes lineage. The *OPB54* MAG encodes nearly 40 CAZyme sequences predicted to act on plant polysaccharides, and compared to most abundant MAGs in the cellulose-fed metagenome, it comprised a high number of predicted pectinolytic enzymes. Specifically, the *OPB54* MAG uniquely encodes sequences belonging to families PL22 (oligogalacturonan lyases) and PL26 (rhamnogalacturonan lyases), and 50% of sequences belonging to family GH28. *TG3* was identified in the pretreated poplar-fed metagenome, which assigned *TG3* to the *Fibrobacterota* phylum (TGPmsp_mb_bin.17). The *TG3* MAG encodes 44 CAZyme sequences predicted to act on plant polysaccharides, with a distribution close to that observed for the corresponding metagenome (**Figure 4A**, **Table S8**).

Members of the archaeal phylum *Bathyarchaeota* are found in many anoxic environments and reportedly transform both plant polysaccharides and lignin (Zhou et al., 2018). Several MAGs belonging to the *Bathyarchaeota* were identified in the metagenome sequences assembled herein; however, only one MAG (TGPidbaud_mb_bin.66) encoded a CAZyme predicted to act on plant-derived carbohydrates (belonging to family GH2; **Tables S6**, **S8**).

### Prediction of polysaccharide utilization loci

**Figure 5:**
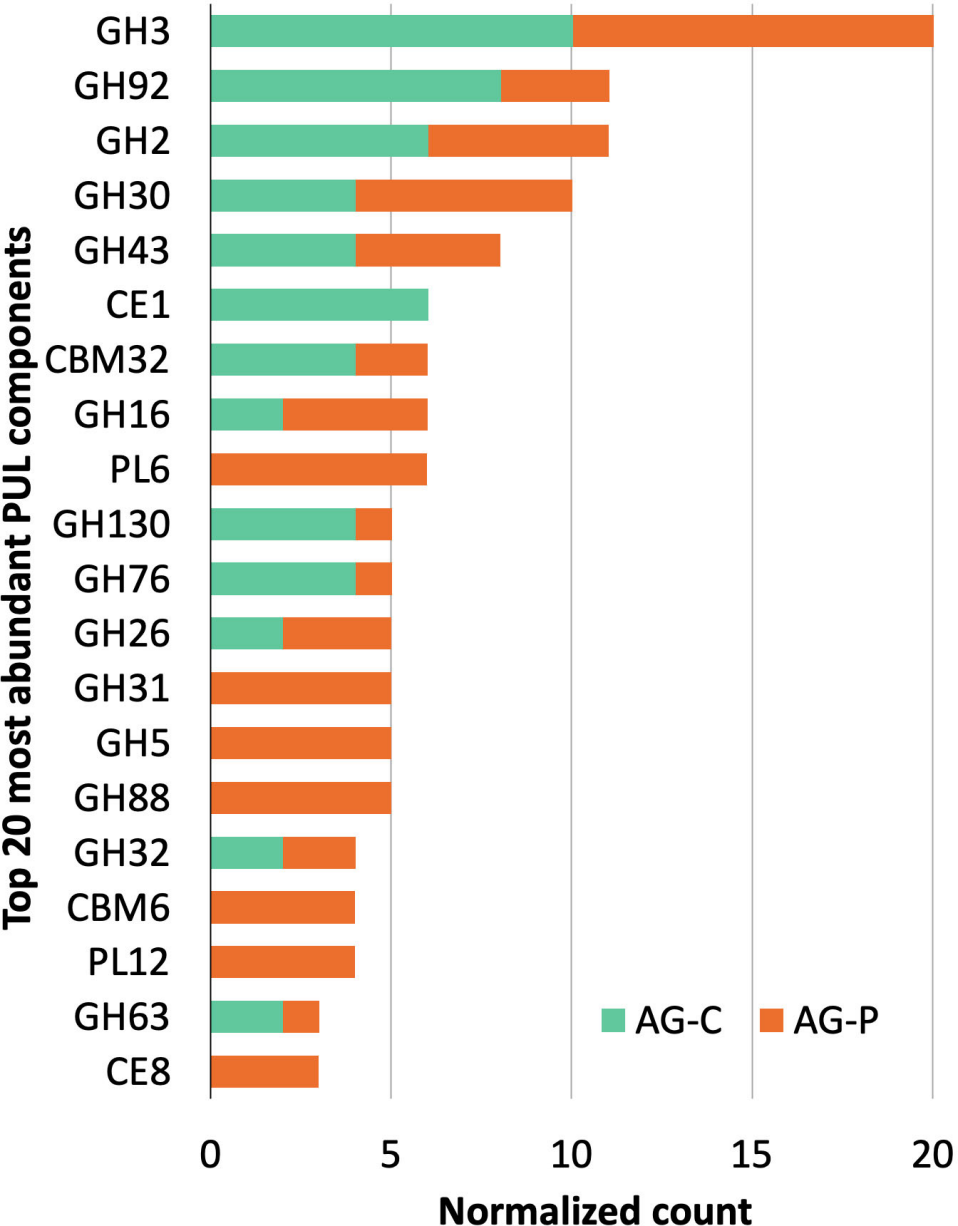
Top 20 most abundant CAZyme families identified in predicted PULs from cellulose- and pretreated poplar-fed pulp mill anaerobic granules microcosms.

As a specialized adaptation for polysaccharide degradation, genomes of many gram-negative bacteria within the *Bacteroidetes, Gemmatimonadetes, Ignavibacteriae, Gemmatimon, Balneolaeota* phyla feature PULs that comprise physically-linked genes that encode CAZymes for stepwise binding, hydrolysis and uptake of plant polysaccharides (Terrapon et al., 2018). Of the 800 sequences from cellulose- and pretreated poplar-fed enrichments that were predicted to encode CAZymes targeting plant polysaccharides, over 25% were present in identified PULs. Consistent with the overall distribution of predicted CAZyme sequences, those belonging to families GH3, GH2, and GH43 were most frequently identified in the predicted PULs (**Figure 5**). The PULs were identified in eight MAGs, where seven were classified as belonging to the phylum *Bacteroidota* and one containing a single two-gene PUL was classified as belonging to *Cloacimonas acidaminovorans* (TGP_mgh_maxbin.006) (**Table S6**). Whereas three of the PUL-containing MAGs were detected in the cellulose-fed enrichment, including the highly abundant *Parabacteroides* MAG (TG_idbaud_maxbin.144), all eight were detected in the pretreated poplar-fed enrichment with the *Paludibacteraceae* MAG (TGP_msp_maxbin.009) and the *Ignavibacteria* MAG (TGP_idbaud_maxbin.007l) being the most enriched (**Table S5**).

## Conclusion

Following cultivation on cellulose and pretreated poplar, microbial communities originating from anaerobic pulp mill digesters encoded a similar profile of predicted plant-polysaccharide active enzymes to communities originating from animal digestive systems (Wong et al., 2017). This observation underscores the considerable impact that substrate amendment has on the functional potential of microbial communities originating from disparate environmental sources. The metagenome assembled genomes of lignocellulose-degrading microbial communities completed herein highlight that OTU abundance does not always identify microorganisms that encode the highest complement of enzymes needed to access the amended lignocellulosic substrate. This was especially apparent for microbial communities enriched on pretreated poplar wood chips. Interestingly, most of the CAZymes predicted to act on plant polysaccharides were found in a small subset of the MAGs constructed. For example, 4 MAGs from the cellulose-fed enrichment encode 219 of the 245 CAZymes predicted to act on plant polysaccharides, while 8 MAGs from the pretreated poplar-fed enrichment encode 403 of the 555 predicted CAZymes. This suggests that a small selection of organisms directly participates in the plant polysaccharide deconstruction and supports the rest of the community. Lastly, the reported MAG analyses shed light on the functional contributions of OTUs that reoccur in lignocellulose-degrading communities (e.g., *TG3* and *OPB54*), which can inform the selection of new CAZyme sequences for functional characterization.

## Data availability statement

The datasets presented in this study can be found in online repositories. The names of the repository/repositories and accession number(s) can be found in the article/**Supplementary Material**.

## Author contributions

MW performed the sequence analyses, data interpretation, and compiled the manuscript. CN generated the metagenome assembled genomes and MAG analyses. WW maintained the enrichment cultures, prepared DNA samples for sequencing. MC contributed to data interpretation. BH, NT, VL and PL contributed to the search and annotation of CAZyme modules and PULs, as well as data interpretation. EM and EE conceived and coordinated the study. All authors contributed to the article and approved the submitted version.
