# Supplementary material for: Taxonomic composition and carbohydrate-active enzyme content in microbial enrichments from pulp mill anaerobic granules after cultivation on lignocellulosic substrates
Source: Front Microbiomes. 2023 Sep 27;2:1094865. doi: 10.3389/frmbi.2023.1094865 (PMC12993600; doi:10.3389/frmbi.2023.1094865)
Supplement: Supplementary file 1 [file DataSheet_1.zip › List of Supplementary Tables.DOCX]

# SUPPLEMENTAL TABLES (List)

**Table S1.** Amplicon DNA concentration, numbers of reads and OTUs for amplicon samples prepared from the pulp mill anaerobic granules and the enrichment microcosms

**Table S2.** a) OTU table summarizing the results from OTU clustering using QIIME and taxonomic assignment by comparison to the Greengenes database; b) Summary of taxonomic assignment of 16S rRNA amplicon data with OTUs aggregated at the order level. Please see Table S2 excel file.

**Table S3.** Results from metagenome assemblies. Three assemblers were used; megahit (Li *et al.*, 2015), metaspades (Nurk *et al.*, 2017) and idba-ud (Peng *et al.*, 2012).

**Table S4.** Overview of MAGs reconstructed from the TGC and TGP metagenomes. Statistics for each MAG obtained using CheckM are reported in the columns with yellow heading. Statistics obtained from Anvi'o are given in the columns labelled in blue. Please see the Table S4 excel file. The FASTA files of all the MAGS are available at: <https://doi.org/10.6084/m9.figshare.21082585.v1>

**Table S5.** All carbohydrate-active enzyme (CAZyme) assignments predicted in metagenomes from anaerobic granules enriched on cellulose (AG-C) or pretreated poplar (AG-P): <https://figshare.com/articles/dataset/Predicted_CAZymes_from_two_metagenomes/21082033>

**Table S6.** Number of CAZyme sequences predicted to act on plant polysaccharides encoded in metagenome assembled genomes (MAGs): <https://doi.org/10.6084/m9.figshare.21082033.v1>

**Table S7.** Distribution of CAZyme sequences predicted to act on plant polysaccharides encoded in metagenome assembled genomes (MAGs) from the cellulose-fed enrichment culture. Please see Table S7 excel file.

**Table S8.** Distribution of CAZyme sequences predicted to act on plant polysaccharides encoded in metagenome assembled genomes (MAGs) from the pretreated poplar-fed enrichment culture. Please see Table S8 excel file.
