# Supplementary material for: Taxonomic composition and carbohydrate-active enzyme content in microbial enrichments from pulp mill anaerobic granules after cultivation on lignocellulosic substrates
Source: Front Microbiomes. 2023 Sep 27;2:1094865. doi: 10.3389/frmbi.2023.1094865 (PMC12993600; doi:10.3389/frmbi.2023.1094865)
Supplement: Supplementary file 1 [file DataSheet_1.zip › Supplementary Figures.DOCX]

## SUPPLEMENTAL FIGURES

**Figure S1.** Chao1 indices, Phylogenetic Diversity (whole tree) and number of observed species of the pulp mill anaerobic granule inocula and the corresponding enrichments. Error bars indicate standard deviation; n=3.

**Figure S2.** Taxonomic composition (order level) of metagenomic reads based on rRNA reads extracted from the libraries.
