# Supplementary material for: Taxonomic composition and carbohydrate-active enzyme content in microbial enrichments from pulp mill anaerobic granules after cultivation on lignocellulosic substrates
Source: Front Microbiomes. 2023 Sep 27;2:1094865. doi: 10.3389/frmbi.2023.1094865 (PMC12993600; doi:10.3389/frmbi.2023.1094865)
Supplement: Supplementary file 1 [file DataSheet_1.zip › Table S1.DOCX]

**Table S1.** Amplicon DNA concentration, numbers of reads and OTUs for amplicon samples prepared from the pulp mill anaerobic granules and the enrichment microcosms

| Sample | Replicates | Amplicon DNA  concentration (µg/ml) | Number of reads | Number of OTUs |
| --- | --- | --- | --- | --- |
| Inoculum | 1 | 106.8 | 3921 | 963 |
|  | 2 | 71.4 | 4546 | 1030 |
|  | 3 | 107.8 | 4594 | 1020 |
| Cellulose | 1 | 146.7 | 7379 | 533 |
|  | 2 | 154.8 | 7038 | 460 |
|  | 3 | 205.4 | 9463 | 561 |
| Cellulose + Lignosulphonate | 1 | 137.1 | 7140 | 568 |
|  | 2 | 139.3 | 6478 | 479 |
|  | 3 | 118.3 | 5808 | 412 |
| Cellulose + Tannic acid | 1 | 68.8 | 7742 | 852 |
|  | 2 | 60.5 | 7347 | 602 |
|  | 3 | 76.4 | 7534 | 631 |
| Pretreated poplar | 1 | 34.6 | 5320 | 838 |
|  | 2 | 60.1 | 8562 | 1130 |
|  | 3 | 58.0 | 6346 | 940 |
