# Supplementary material for: Taxonomic composition and carbohydrate-active enzyme content in microbial enrichments from pulp mill anaerobic granules after cultivation on lignocellulosic substrates
Source: Front Microbiomes. 2023 Sep 27;2:1094865. doi: 10.3389/frmbi.2023.1094865 (PMC12993600; doi:10.3389/frmbi.2023.1094865)
Supplement: Supplementary file 1 [file DataSheet_1.zip › Table S3.DOCX]

**Table S3.** Results from metagenome assemblies. Three assemblers were used; megahit (Li *et al.*, 2015), metaspades (Nurk *et al.*, 2017) and idba-ud (Peng *et al.*, 2012).

| Metagenome | assembly name | assembler | size (bp) | no contigs | N50 | longest contig | no  genomes^a^ |
| --- | --- | --- | --- | --- | --- | --- | --- |
| AG-C | TGCmsp | metaspades | 222,482,845 | 46,379 | 14,397 | 1,113,541 | 56 / 7 |
| AG-C | TGCidbaud | idba-ud | 180,028,085 | 30,862 | 15,500 | 732,750 | 49 / 6 |
| AG-C | TGCmgh | megahit | 216,604,701 | 44,572 | 12,966 | 1,290,127 | 56 / 6 |
| AG-P | TGPmsp | metaspades | 526,456,694 | 130,470 | 10,166 | 1,240,148 | 163 / 25 |
| AG-P | TGPidbaud | idba-ud | 405,601,308 | 78,120 | 14,245 | 481,921 | 122 / 20 |
| AG-P | TGPmgh | megahit | 511,916,877 | 124,560 | 10,083 | 576,025 | 124 / 27 |
| AG-C+AG-P | TGmsp | metaspades | 695,504,175 | 169,175 | 10,513 | 1,240,148 | 171/ 28 |
| AG-C+AG-P | TGidba-ud | idba-ud | 531,864,903 | 104,026 | 12,837 | 697,857 | 125 / 21 |
| AG-C+AG-P | TGmgh | megahit | 675,114,122 | 161,879 | 9,981 | 698,053 | 181 / 30 |

^a^ Number of bacterial genomes / number of archaeal genomes, estimated from single copy genes in Anvi’o ver. 6.2. AG-C (anaerobic granule grown on cellulose); AG-P (anaerobic granules grown pretreated poplar wood chips)
